# High Loading of Hydrophobic and Hydrophilic Agents via Small Immunostimulatory Carrier for Enhanced Tumor Penetration and Combinational Therapy

**DOI:** 10.7150/thno.38287

**Published:** 2020-01-01

**Authors:** Jingjing Sun, Yichao Chen, Jieni Xu, Xiangping Song, Zhuoya Wan, Yuqian Du, Weina Ma, Xizhen Li, Lin Zhang, Song Li

**Affiliations:** 1 Center for Pharmacogenetics, University of Pittsburgh, Pittsburgh, PA, 15261, USA.; 2 Department of Pharmaceutical Sciences, School of Pharmacy, University of Pittsburgh, Pittsburgh, PA, 15261, USA.; 3 UPMC Hillman Cancer Center, University of Pittsburgh, Pittsburgh, PA, 15261, USA.; 4 Department of Pharmacology & Chemical Biology, University of Pittsburgh, Pittsburgh, PA, 15261, USA.

**Keywords:** tumor penetration, high loading capacity, T-cell immune response, patient derived xenograft (PDX)

## Abstract

Development of small-sized nanoformulations for effective tumor penetration, particularly for those tumors with dense stroma is a major challenge in cancer nanomedicine. It is even more challenging to achieve effective co-loading of both hydrophobic and hydrophilic anticancer agents through a small-sized nanocarrier. In this work, we designed a novel redox-responsive gemcitabine (GEM)-conjugated polymer POEG-co-PVDGEM (PGEM) as a small-sized nanocarrier to co-deliver hydrophilic GEM and hydrophobic paclitaxel (PTX).

**Methods:** The *in vitro* physicochemical and biological properties of PTX/PGEM NPs were characterized. The efficiency of the PGEM carrier in selective codelivery of GEM and PTX in two murine tumor models as well as a patient derived xenograft model (PDX) was also evaluated. In addition, we investigated the changes in tumor immune microenvironment after treatment with PTX/PGEM nanoparticles.

**Results:** We discovered that GEM conjugation could significantly decrease the nanoparticle size from 160 nm to 13 nm. Moreover, different from most reported GEM-conjugated polymers, PGEM polymer could serve as a prodrug carrier to load a wide variety of hydrophobic agents with high drug loading capacity and excellent stability. More importantly, our strategy could be extended to various nucleotides-based drugs such as azacytidine, decitabine and cytarabine, suggesting a new platform for co-delivery of various first line hydrophilic and hydrophobic anticancer agents. Imaging showed that our small-sized carrier was much more effective in tumor accumulation and penetration compared to the relatively large-sized drug carrier. The PGEM prodrug-based carrier not only well retained the pharmacological activity of GEM, but also boosted T-cell immune response. Furthermore, delivery of PTX via PGEM led to significantly improved antitumor activity in several murine cancer models and a PDX model of colon cancer.

**Conclusion:** This work not only provided a small-sized carrier platform that was able to load multiple hydrophilic and hydrophobic drugs with high loading capacity, but also provided an effective regimen for enhanced tumor penetration and improved anti-tumor immunity.

## Introduction

Nanomedicine, which utilizes nanoparticles to deliver therapeutic agents, offers numerous benefits in treating cancers, such as increasing drug solubility, improving the drug accumulation in the tumors, and reducing the toxicity. During the design of an intravenously injectable nanomedicine, particle size is a key physicochemical parameter to be considered due to its vital role in the drug loading, biodistribution and tumor penetration. It has been generally regarded that particles of 4-200 nm are capable of selectively accumulating in tumors due to the leaky vessels in tumors 1-5; however, the vessel pores of many tumor types, such as pancreatic and breast cancers, have much smaller pore cutoff sizes (~50-60 nm) 6. Accordingly, the nanoparticles of less than 60 nm are preferable to achieve effective extravasation and deep penetration in these tumor tissues. This has also been confirmed in recent studies 7-9. For example, Cabral et al prepared several 1,2-diaminocyclohexane-platinum(II) loaded nanoparticles with different sizes (30, 50, 70 and 100 nm), and found only nanoparticles smaller than 50 nm could penetrate pancreatic tumors 7. Similarly, Chauhan et al 10 and Huang et al 11 reported that nanoparticles of small sizes (~10 nm) exhibited superior tumor penetration compared to larger nanoparticles in 4T1 breast tumor model. Recently considerable efforts have been made in developing smaller nanoparticles (NPs) for effective tumor penetration but these NPs often have limited effectiveness in drug loading, particularly for simultaneous co-loading of hydrophobic and hydrophilic drugs 12-15.

Gemcitabine (GEM) is a water-soluble chemotherapeutic drug for the treatment of various malignant tumors 16, 17. In addition to its direct cytotoxic effect on tumor cells, GEM has demonstrated the capability to stimulate the immune system against cancer 18, 19. However, limited clinical benefits were achieved because of its very short half-life in blood and rapid inactivation by cytidine deaminase (CDA) 20. To improve the treatment outcome, combinations of GEM with other chemotherapies or targeted therapies (such as nab‑paclitaxel and erlotinib) have been evaluated in preclinical and clinical studies. However, due to their distinct physical properties, it is difficult to co-deliver GEM and other hydrophobic agents at their optimal dosages to tumors, especially using a small nanocarrier for penetrating the dense stroma to reach the tumor core.

**Figure 1:**
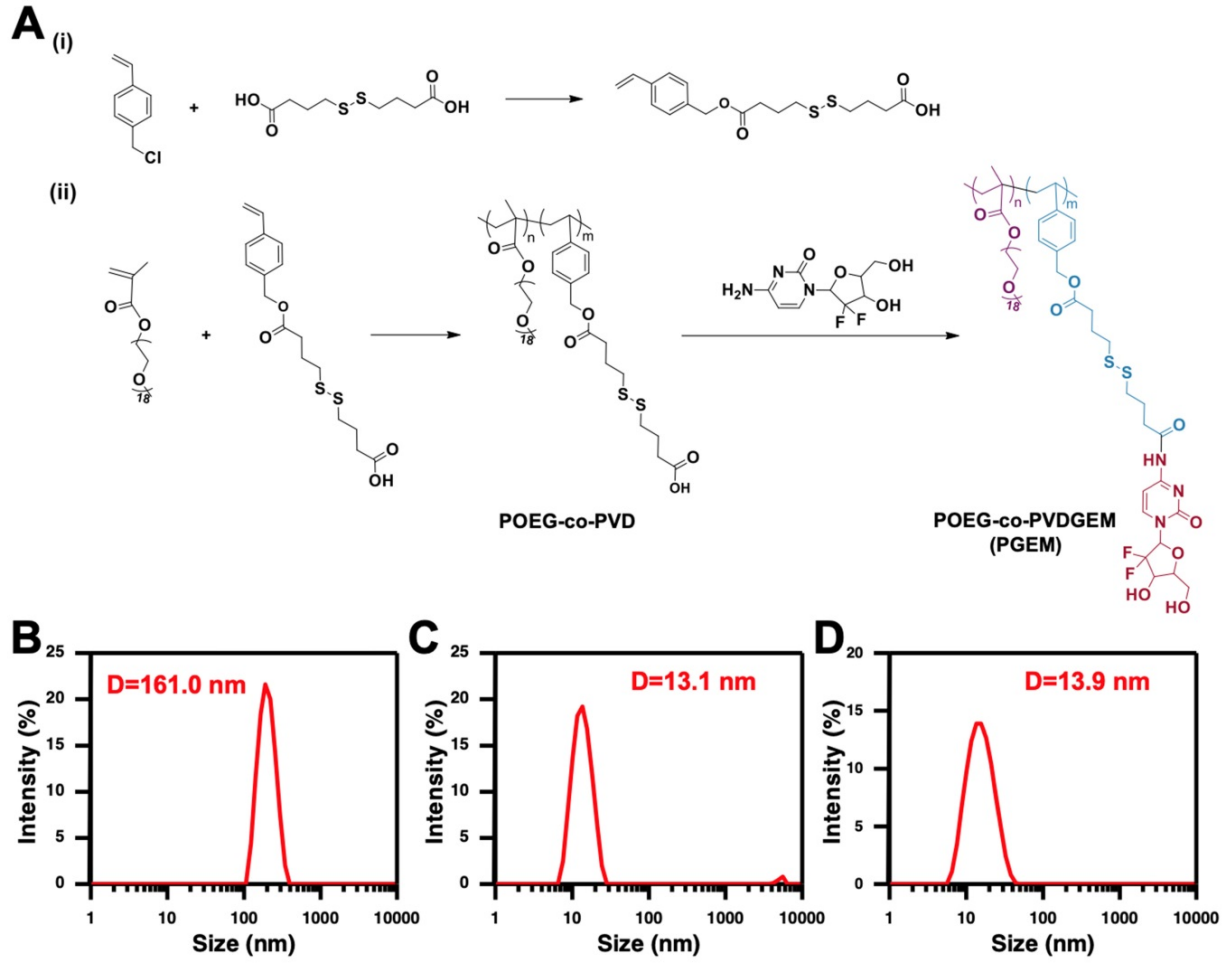
** (A)** Synthesis routes of the PGEM polymers via RAFT polymerization and subsequent conjugation with GEM. The size distribution of POEG-*co*-PVD **(B)**, PGEM **(C)** and PTX-loaded PGEM micelles **(D)** by DLS.

Herein, we reported a small sized nanocarrier assembled from redox-responsive GEM-conjugated polymer POEG-co-PVDGEM (PGEM) (**Figure 1A**). We discovered that GEM conjugation drastically decreased the nanoparticle size from 160 nm to 13 nm, leading to significantly improved accumulation and penetration in the tumors. More interestingly, the small PGEM carrier was highly effective in loading various types of hydrophobic drugs (~24% w/w DLC). PTX was chosen as a model drug in this work because of its wide application in the clinical treatment of various solid tumors such as pancreatic, breast, colon and lung cancers 21, 22. In addition, PTX and GEM have been reported to show synergy in the overall antitumor activity 23, 24. We evaluated the efficiency of the PGEM carrier in selective codelivery of GEM and PTX in two murine tumor models as well as a patient derived xenograft model (PDX). In addition, we investigated the changes in tumor immune microenvironment after treatment with PTX/PGEM nanoparticles.

## Materials and methods

### Materials

Vinylbenzyl chloride, 4,4'-Dithiodibutyric acid, oligo(ethylene glycol) methacrylate (OEG950 monomer, average M*_n_*=950), 4-Cyano-4-(phenylcarbonothioylthio)pentanoic acid, 2, 2-Azobis(isobutyronitrile) (AIBN), trypsin-EDTA solution, 3-(4,5-dimethylthiazol-2-yl)-2,5-diphenyl tetrazolium bromide (MTT) and Dulbecco's Modified Eagle's Medium (DMEM) were all bought from Sigma-Aldrich (MO, U. S. A.). AIBN was purified by recrystallization in anhydrous ethanol. 1-hydroxybenzotriazole (HOBT) and 1-(3-dimethylaminopropyl)-3-ethylcarbodiimide HCl (EDC) were purchased from GL Biochem (Shanghai, China). Diisopropylethylamine (DIPEA) was purchased from Acros Organics. Paclitaxel was purchased from AK Scientific Inc. (CA, U. S. A.). Doxorubicin hydrochloride salt (DOX·HCl) and gemcitabine (GEM) were purchased from LC Laboratories (MA, USA). Fetal bovine serum (FBS) and penicillin-streptomycin solution were purchased from Invitrogen (NY, U. S. A.). The animal-related experiments were performed in full compliance with institutional guidelines and approved by the Animal Use and Care Administrative Advisory Committee at the University of Pittsburgh. Informed consent was obtained for experimentation with patient-derived xenograft (PDX) model. And the PDX-related experiments were carried out in accordance with The Code of Ethics of the World Medical Association.

### Characterization

The structures of monomer and polymers were characterized by ^1^H NMR spectrum on a Varian 400 FT-NMR spectrometer (400.0 MHz). FTIR spectra were recorded on a Bio-Rad FTS-185 spectrometer at room temperature. The molecular weight (*M*_n_ and *M*_w_) and polydispersity index (*M*_w_/*M*_n_) of polymers were determined by gel permeation chromatography (GPC) with a Waters 2414 refractive index detector. A series of commercial polystyrene standards were used for calibration curves. The average particle size, size distribution and morphology of micelles were measured by dynamic light scattering (DLS, Malvern Zeta Sizer) and transmission electron microscopy (TEM).

### Synthesis of VD monomer

Vinylbenzyl chloride (305.2 mg, 2 mM), 4,4'-Dithiodibutyric acid (2.38 g, 10 mM) and K_2_CO_3_ (0.69 g, 5 mM) were dissolved in 10 mL DMF and reacted at 50°C under stirring. After 16 h, the mixture was cooled down to room temperature, followed by adding 80 mL of CH_2_Cl_2_. The mixture was centrifuged at 4500 rpm for 12 min and the supernatant was washed with water for three times, and then dried with anhydrous sodium sulfate. The VD monomer was obtained by column chromatography purification with ethyl acetate/petroleum ether (v/v, 1/2~1/1) as the elution.

### Synthesis of POEG-*co*-PVD polymer

4-Cyano-4-(thiobenzoylthio)pentanoic acid (6 mg, 0.0215 mmol), AIBN (2 mg, 0.0124 mmol), VD monomer (300 mg, 0.95 mmol), OEG950 monomer (400 mg, 0.42 mmol), and 2 mL of dried tetrahydrofuran were added into a Schlenk tube. After deoxygenation with three free-pump-thawing cycles, the mixture was stirred at 80 °C under the protection of N_2_ for 18 h. Then, the reaction was quenched by liquid nitrogen and the POEG-*co*-PVD polymer was obtained by precipitation in ether for 3 times. Conversion_(OEG950 monomer)_ = 45.9%; Conversion_(VD monomer)_ = 50.0%.

### Synthesis of PGEM polymer

The as-synthesized POEG-*co*-PVD polymer (120 mg, 0.17 mmol -COOH), GEM (179 mg, 0.68 mmol), HOBT (270 mg, 2 mmol), and EDC (450 mg, 2.35 mmol) were dissolved in 30 mL of DMSO with the addition of 300 μL of DIPEA. After stirring at 37 °C for 72 h, the mixture was dialyzed against DMSO and then water for 2~3 days. The PGEM polymer was obtained after lyophilization.

### Determination of GEM loading content in PGEM polymer

The GEM loading content in PGEM polymer was quantified by alkaline hydrolysis method with 1 N NaOH 25, 26. The amount of GEM in the polymer was measured by high performance liquid chromatography (HPLC) with UV detector at the wavelength of 268 nm using methanol/water (04:96 v/v) as a mobile phase.

### Preparation and characterization of drug-loaded micelles

Blank and drug-loaded micelles were prepared by film hydration method 27. Briefly, PGEM polymer and anti-cancer drugs were mixed in dichloromethane/methanol with different carrier/drug ratios. After completely removing the organic solvents, a thin film was formed, which was then hydrated with PBS solution to give PTX-loaded PGEM micelles.

Drug loading capacity (DLC) and drug loading efficiency (DLE) were determined by HPLC and calculated according to the following equations:

DLC (%) = [weight of drug loaded/(weight of polymer + drug used)] ×100

DLE (%) = (weight of loaded drug/weight of input drug) × 100

### Critical micelle concentration (CMC) of PGEM micelles

The CMC value of PGEM micelles was measured using nile red as a fluorescence probe 28. PGEM micelles (1 mg/mL) were prepared by film hydration method, and diluted into different concentrations, which were then added to each vial containing nile red. After overnight incubation, fluorescence intensities of the solutions were measured by fluorescence spectrometer.

### Stability of PTX/PGEM NPs

PTX/PGEM NPs were incubated in the FBS-containing PBS solution (50% FBS), and the size changes were monitored by DLS over time with PTX/PGEM NPs (without FBS) as a control.

Redox-responsive destabilization of PTX/PGEM NPs was observed by detecting the size changes in the absence/presence of 10 mM GSH. The NPs solution was kept in a shaking bath at 37 °C for 24 h before measurement.

### Release of PTX and GEM from PTX/PGEM NPs

Briefly, 0.5 mL of PTX/PGEM NPs were placed in a dialysis bag (MWCO 3.5 kDa) and immersed in 40 mL of 0.1 M PBS buffer solution containing 0.5% (w/v) Tween 80 or/and 10 mM GSH or 10% FBS. The experiment was performed in an incubation shaker at 37 °C at 150 rpm. At selected time intervals, the PTX concentration in the dialysis bag was tested by HPLC at 227 nm wavelength. The conjugated GEM concentration in the dialysis bag was tested by alkaline hydrolysis method with 1 N NaOH, followed by HPLC detection 25, 26. GEM and PTX release from Taxol plus GEM group was included as the control.

### MTT assay

The cytotoxicity of blank and drug-loaded micelles was investigated by MTT assay using murine pancreatic carcinoma cell lines PANC02 and H7. Cells were seeded into a 96-well plate at a density of 5000 cells/well and incubated in 100 μL of Dulbecco's Modified Eagle medium (DMEM) containing 10% FBS for 24 h. Cells were treated with various concentrations of micellar formulations and Taxol plus GEM for 72 h. Then, 50 μL of MTT solution (2 mg/mL) were added to each well and the cells were incubated for another 4 h. After removing the medium, 100 μL of DMSO were added into each well to dissolve MTT formazan crystals. The optical density was measured using a microplate reader and the cell viability was calculated with untreated cells as a control.

The combinational effect of PTX and GEM was similarly evaluated. Cells were treated with PTX and GEM at various concentrations for 96 h. The cell viabilities were determined by using MTT method. The combination index (CI) was calculated using the formula: CI = C_PTX, X_/IC_X_, _PTX_ + C_GEM, X_/IC_X_, _GEM_.

Where C_PTX_ and C_GEM_ are the concentrations of PTX and GEM used in combination to achieve X% inhibition effect. IC_X_, _PTX_ and IC_X, GEM_ are the concentrations for single agents to achieve X% inhibition effect. CI <1, =1 and >1 indicate synergic, additive or antagonistic effect, respectively.

### *In vitro* hemolysis assay

Red blood cells were collected by centrifugation of fresh rat blood at 700 g for 10 min, and then washed with cold PBS for three times. The PBS suspensions of red blood cells were treated with POEG-*co*-PVD, PGEM, branched PEI25K, Triton X-100 (2%, positive controls) and PBS (negative control), respectively. After incubation in a shaker at 37°C for 60 min, the mixtures were centrifuged, and the supernatants were transferred into a 96-well plate. The release of hemoglobin was determined at 540 nm, and calculated as (OD_sample_ - OD_negative control_)/(OD_positive control_ - OD_negative control_) × 100%.

### Cellular uptake

PANC02 cells were seeded in a 12-well plate at a density of 1 × 10^5^ cells/well. Following culture for 24 h, cells were treated with free Rhodamine, Rhodamine/PGEM and Rhodamine/POEG-*co*-PVD micelles in FBS-free culture medium (Life Technologies, USA) for 4 h, respectively. The concentration of Rhodamine was kept at 1 μg/mL. Then cells were stained with Hoechst 33342 (1 mg/mL) for 20 min, and washed with ice-cold PBS. The cellular distribution was observed under a fluorescence microscope (BZ-X710, Japan).

### Blood circulation, tissue biodistribution and penetration

DiR-loaded POEG-*co*-PVD micelles and PGEM micelles with a DiR concentration of 0.2 mg/mL were injected into C57BL/6 mice. At indicated time points, blood samples were collected and centrifuged at 8000 rpm for 10 min. The supernatants were collected and imaged by IVIS 200 system (Perkin Elmer, USA) at a 60 s exposure time with excitation at 730 nm and emission at 835 nm.

For tissue distribution study, the PDX tumor-bearing mice (~1500 mm^3^) were injected with free DiR, DiR-loaded POEG-co-PVD micelles and PGEM micelles. The mice were sacrificed at 24 h, and the major organs were excised for *ex vivo* imaging by IVIS 200 system. Afterwards, the tumors were frozen sectioned at 10-μm thickness and stained with DAPI to label the cell nucleus. The fluorescence signals were examined under a fluorescence microscope (BZ-X710, Japan).

Tumor penetration study was also performed in PANC02-bearing mice. To minimize any individual differences in tumors, fluorescence probes rhodamine and fluorescein were loaded into PGEM carrier and POEG-*co*-PVD carrier, respectively 29. These nanoparticles (4 mg each) were mixed in 200 μL PBS and co-injected into the mice via the tail vein. Tumor sections were collected at 15 h and co-stained with DAPI. The fluorescence signals were examined under a fluorescence microscope (OLYMPUS America, Melville, NY). In a parallel study, the fluorescence probes were switched and fluorescein and rhodamine were loaded into PGEM carrier and POEG-*co*-PVD carrier, respectively. Similarly, the mixed nanoparticles were co-injected into the mice for imaging study.

### *In vivo* efficacy in PANC02 model

A syngeneic PANC02 pancreatic tumor model was established by inoculating 2×10^5^ PANC02 cells into the flank of C57BL/6 mice. When the tumor volume reached around 50 mm^3^, mice were divided into six groups (n=5) and treated with PBS, PGEM micelles, PTX/PGEM micelles, and combination of Taxol and free GEM, respectively, every three days for a total of 5 times. The dosage of GEM and PTX were kept at 20 mg/kg and 10 mg/kg. Tumor volumes and mouse body weights were measured every three days. The tumor volumes (V) were calculated by the formula: V= (length of tumor) × (width of tumor)^2^/2. After the completion of the experiment, tumor tissues were excised and fixed with 10% formaldehyde, followed by embedment in paraffin. The sliced tissues at 5 µm were stained by hematoxylin and eosin (H&E) and observed under a Zeiss Axiostar Plus microscope (PA, USA).

### Quantification of tumor-infiltrating lymphocytes by flow cytometry

C57BL/6 mice bearing PANC02 tumors received various treatments via i.v. administration every 3 days for 3 times. Tumors and spleens were excised at 24 h following the last treatment. Single cell suspensions were filtered and red blood cells were lysed. Then the cells were stained with various antibodies for flow cytometry analysis with FlowJo software (Tree Star Inc.) 30.

### Efficacy study in CT26 and PDX models

CT26 colon cancer model was established by s.c. inoculating 1×10^6^ CT26 cells into right flank of the BALB/c-J mice. The mice were intravenously injected with various formulations when the tumor volume reached ~100 mm^3^. PDX model was established by s.c. implanting the KRAS-mutant (G13D), NRAS-mutant (G12D), and MMR-proficient tumor (T4N0M1) from the sigmoid colon of a 77-year-old male into both flanks of NSG mice 31. Tumors were passaged for two generations before treatment.

### Statistical analysis

Data are presented as mean ± standard deviation (SD). The differences between groups were compared by one-way analysis of variance (ANOVA), and p < 0.05 is considered statistically significant.

## Results

### Synthesis and characterization of the PGEM polymer

The synthesis route of PGEM polymer was shown in **Figure 1A**. First, vinyl benzyl monomer with disulfide linkage (VD monomer) was synthesized via reaction of vinylbenzyl chloride and 4, 4'-dithiodibutyric acid. Then, POEG-*co*-PVD polymer was synthesized by RAFT co-polymerization of VD monomer and OEG950 monomer. PGEM polymer was finally obtained by conjugating GEM to the POEG-*co*-PVD polymer backbone using EDC/HOBt coupling reaction.

The structures of VD monomer, POEG-*co*-PVD and PGEM polymers were characterized by ^1^H NMR (**Figure S1~S3**). For POEG-*co*-PVD polymer, the average degree of polymerization (DP) of the OEG950 monomer was calculated to be 9 according to the conversion of OEG950 monomer at the end of the polymerization. The DP of the VD monomer was determined to be 23 by comparing the intensities of *I*_c_ and *I*_d_ (**Figure S2**). After conjugation of GEM to POEG-*co*-PVD polymer, protons peaks corresponding to GEM were observed in the ^1^H NMR spectrum, and the number of GEM units per polymer molecule was determined to be 8 by comparing the intensities of *I*_c_ and *I*_d_ (**Figure S3**). Compared to POEG-*co*-PVD polymer, the appearance of the hydroxyl group signal (*ν*_O-H_) at 3445 cm^-1^ in the FTIR spectrum of PGEM (**Figure S4**) further confirmed the successful conjugation of GEM onto POEG-*co*-PVD backbone. GEM loading capacity was also determined by HPLC-UV analysis via the alkaline hydrolysis method 25, 26. A gemcitabine loading in the PGEM polymeric carrier was determined to be 8.9% w/w.

The molecular weights and distributions of the POEG-*co*-PVD and PGEM polymers were also determined by GPC. As summarized in **Table S1**, the number average molecular weight *M*_n_ determined by GPC is 11600 for POEG-*co*-PVD and 9200 for PGEM, respectively, and both polymers showed low polydispersity of 1.12. It is noted that the *M*_n_ of PGEM determined by the GPC was decreased after GEM conjugation to the POEG-*co*-PVD polymer backbone. It is well known that GPC separates the polymers by hydrodynamic size instead of molar mass. So the decrease in measured polystyrene-relative molecular weight of PGEM indicated a compaction of the polymer chain in THF after GEM conjugation. The GPC and NMR results indicated the successful synthesis of PGEM copolymer with defined and controllable structure.

### Physicochemical characterization of micelles

Both POEG-*co*-PVD and PGEM polymers were able to form nanoparticles in the aqueous solution via a simple film hydration method. POEG-*co*-PVD micelles showed a diameter of 161.0 nm (**Figure 1B**). After GEM conjugation, the PGEM polymer formed smaller nanoparticles with diameter decreased to 13.1 nm. (**Figure 1C**), suggesting that the GEM structure played an important role in forming the small-sized nanoparticles. **Figure S6a&b** showed the TEM images of POEG-*co*-PVD and PGEM micelles. POEG-*co*-PVD showed larger size than PGEM NPs, but its number was smaller than that from DLS measurement, which might be due to that TEM exhibits the size of the dried particle. In addition, the critical micelle concentration of PGEM was evaluated by fluorescence spectrometry using nile red as a probe. As shown in **Figure S5**, the CMC value of PGEM micelles is 0.0072 mg/mL, which shall provide a good colloidal stability after dilution in the blood circulation.

PTX could be loaded into PGEM carrier at various carrier/drug ratios, and all of the PTX/PGEM micelles exhibited very small sizes **(Table 1 & Figure 1D)**. At a carrier/drug ratio of 2.5:1 (w/w), the formulation showed a loading capacity of as high as 24.2%. TEM showed spherical morphologies for both PGEM micelles and PTX/PGEM micelles (**Figure S6**). Besides, the PTX/PGEM micelles were stable in PBS and FBS-containing PBS solutions without significant size changes (**Figure S7**). In addition to PTX, the PGEM carrier was also able to load a variety of other hydrophobic agents including NLG919, curcumin, and doxorubicin. All these formulations showed small sizes (15~23 nm) and high loading capacity (8~ 25%).

The redox-sensitivity of PTX/PGEM micelles was evaluated by monitoring the size changes in the absence/presence of 10 mM GSH (**Figure S8**). After incubation for 24 h, PTX/PGEM micelles didn't show size changes in the absence of 10 mM GSH. However, in the presence of 10 mM GSH, a larger size peak appeared, suggesting that part of disulfide linkages in the PTX/PGEM micelles have been cleaved by GSH. The release of PTX and GEM from PTX/PGEM micelles was also evaluated in pH 7.4, pH 7.4+FBS and pH 7.4+ GSH environment. Compared to Taxol plus GEM group, PTX/PGEM micelles showed slower release of PTX (**Figure S9A**). There is no difference in release of PTX from PTX/PGEM micelles in pH 7.4 and pH 7.4+FBS medium. In the presence of 10 mM GSH, the release of PTX from PTX/PGEM micelles was a little faster with more than 40% of PTX being released within 24 h. The result of GEM release from PTX/PGEM micelles was shown in **Figure S9B**. Due to chemical conjugation of GEM onto the polymer backbone, there is little release of GEM from PTX/PGEM micelles in the pH 7.4 and pH 7.4+FBS medium, while the release of GEM from Taxol plus GEM group was faster with 61% of GEM being released at 24 h. In addition, the release of GEM from PTX/PGEM micelles with 10 mM GSH was higher (~20%) than that without GSH at 24 h. These release data were consistent with the size change profiles in **Figure S8**. The inefficient cleavage under GSH might be due to that most of disulfide linkages were buried in the hydrophobic micelle core, where GSH might be not able to reach effectively. More efficient cleavage of GEM could be achieved *in vivo* due to the combination of various esterase and redox environment in tumors.

### Biological activity of PTX formulated in PGEM carrier

**Figure 2:**
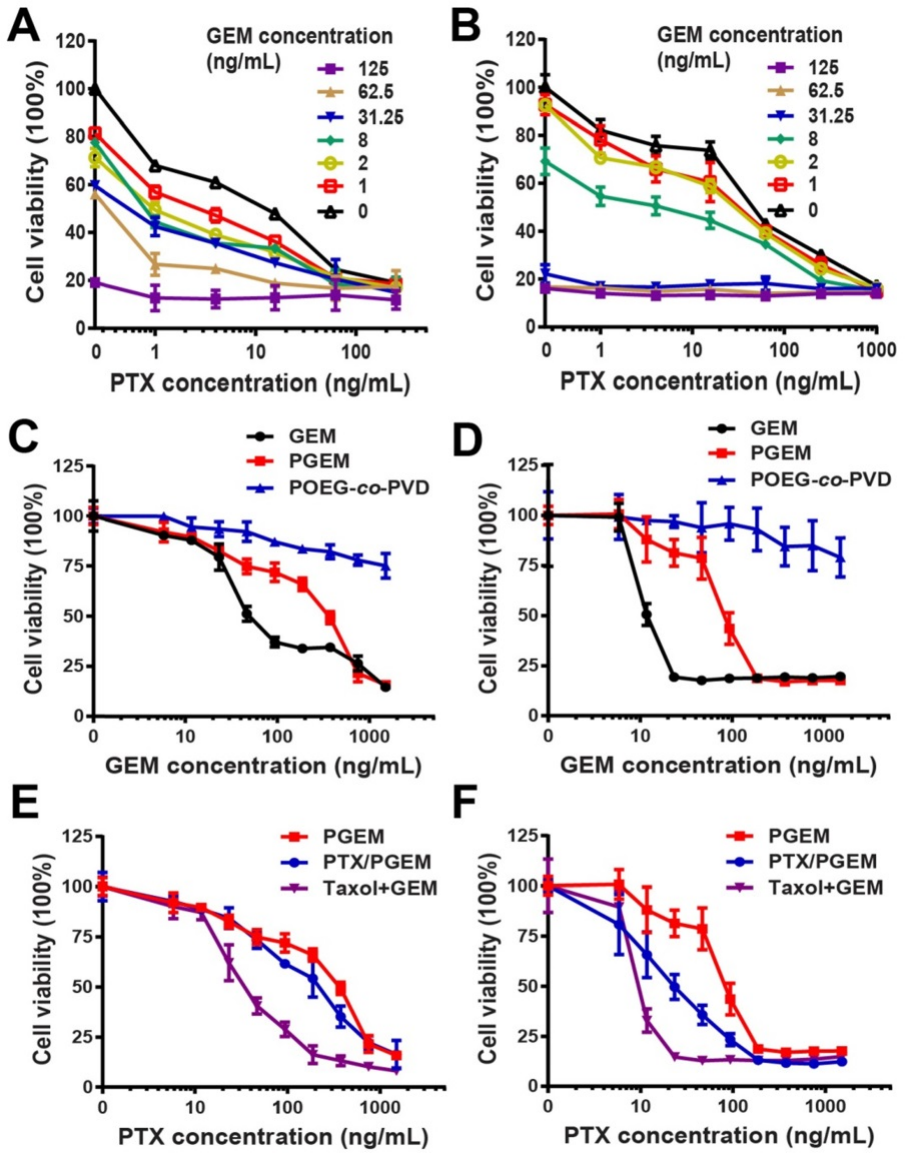
**(A-B)** Proliferation inhibition of PANC02 **(A)** and H7 **(B)** tumor cell lines treated with combination of PTX and GEM for 96 h. Combination index (CI) of PTX and GEM was calculated to 0.5 and 0.6 for PANC02 and H7, respectively. **(C-D)** MTT cytotoxicity assay of PGEM prodrug micelles in PANC02 **(C)** and H7 **(D)** cell lines with free GEM as the control. **(E-F)** MTT cytotoxicity of various formulations in PANC02 **(E)** and H7 **(F)** cells. Cells were treated with different micelles for 72 h. All data are reported as means ± SD.

The combination effect of free GEM and PTX was first examined in pancreatic cancer cell lines PANC02 (**Figure 2A**) and H7 (**Figure 2B**). Compared to single drug, combination of PTX and GEM significantly improved tumor cell killing effect. Combination index (CI) in PANC02 and H7 cells was calculated to be 0.5 and 0.6, respectively, suggesting synergistic effect (CI < 1) of PTX and GEM in both cell lines.

The cytotoxicity of PGEM prodrug micelles was examined in PANC02 (**Figure 2C**) and H7 (**Figure 2D**) cells. It can be seen that both free GEM and PGEM exhibited a concentration-dependent cell killing effect, while POEG-*co*-PVD showed minimal cytotoxicity, suggesting that the cell killing effect of PGEM mainly comes from the conjugated GEM. The IC_50_ of PGEM prodrug micelles was higher than that of free GEM in both PANC02 and H7 cells (**Table S2**). In addition, PTX/PGEM showed higher cytotoxicity than PGEM NPs, but lower cytotoxicity than Taxol and GEM combination group (**Figure 2E&F**). The relatively lower level of cytotoxicity of PGEM and the absence of synergy between PGEM and PTX might be due to the incomplete cleavage of GEM from the PGEM polymer in the cells within the short treatment period.

**Figure S10** shows the hemolysis analysis of POEG-*co*-PVD and PGEM. Branched polyethylenimine (bPEI25K), a well-known polymer with high hemolytic effect was used as a positive control. Compared to bPEI25K, both POEG-*co*-PVD and PGEM showed negligible hemolytic effect with lower hemoglobin release.

Then, the cellular uptake of POEG-*co*-PVD and PGEM NPs were investigated by using rhodamine fluorescence probe. As shown in **Figure S11**, both rhodamine/POEG-*co*-PVD and rhodamine/PGEM NPs were co-localized with cell nucleus after incubation with PANC02 cells for 4 h. There was no significant difference in fluorescence signals between POEG-*co*-PVD and PGEM-treated cells, suggesting similar cellular uptake profiles of POEG-*co*-PVD and PGEM NPs.

### Pharmacokinetics, biodistribution and tumor penetration of PGEM carriers

**Figure 3:**
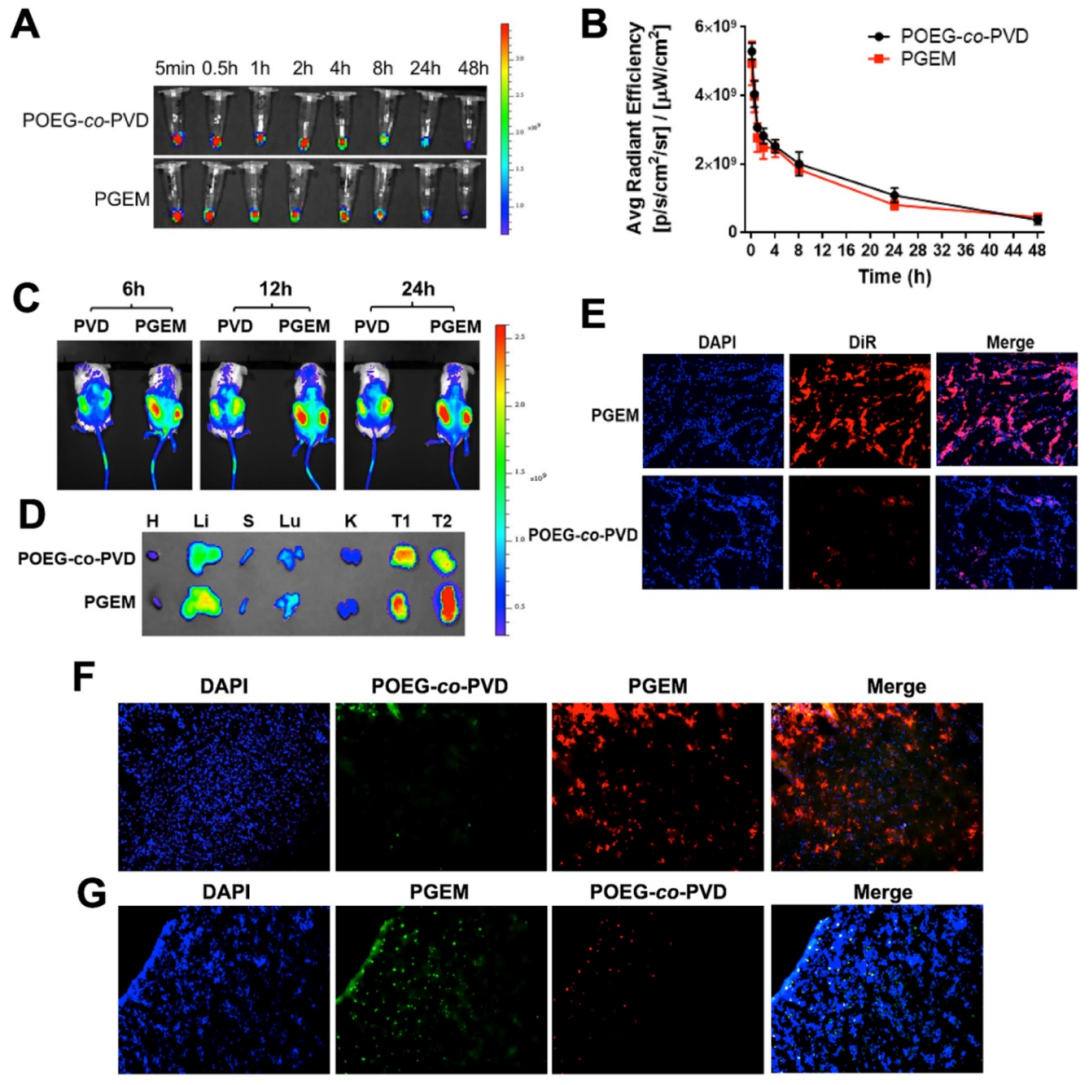
Pharmacokinetics, biodistribution and tumor penetration of PGEM carrier (15 nm NPs) with POEG-*co*-PVD carrier (160 nm NPs) as a control.** (A)** The representative NIR images and **(B)** quantitative measurements of DiR/ PGEM and DiR/POEG-co-PVD in the blood. **(C)** *In vivo* NIR imaging of the PDX tumor-bearing mice treated with DiR-labeled POEG-co-PVD and PGEM micelles at different time points. **(D)** *Ex vivo* NIR images of major organs and tumors of each mouse treated with DiR-labeled POEG-*co*-PVD and PGEM respectively at 24 h. **(E)** Fluorescence images of tumor sections at 24 h after treatment with DiR-loaded POEG-co-PVD and PGEM micelles, respectively. **(F-G)** Fluorescence images of tumor sections at 15 h after treatment with **(F)** the mixture solution of fluorescein-labeled POEG-co-PVD (green, 160 nm NPs) and rhodamine-labeled PGEM (red, 15 nm NPs); **(G)** the mixture solution of fluorescein- labeled PGEM and rhodamine-labeled POEG-co-PVD NPs.

The kinetics of PGEM and POEG-*co*-PVD NPs in blood was investigated by near-infrared fluorescent (NIR) optical imaging using the DiR probe. DiR-loaded PGEM (~13 nm) and POEG-*co*-PVD micelles (~160 nm) were *i.v.* injected into the C57BL/6 mice. **Figure 3A&B** show the representative NIR images and quantitative analysis of fluorescence intensities of NPs in the plasma at different time points after administration. No significant differences in fluorescence intensities were observed for DiR/POEG-co-PVD and DiR/PGEM-treated groups. Both DiR/POEG-*co*-PVD and DiR/PGEM showed a relatively long circulation in the blood. At 24 h, DiR/POEG-co-PVD and DiR/PGEM retained relatively high fluorescence signals (~20%).

Next, we evaluated the biodistribution and penetration of PGEM carrier in a patient-derived xenograft (PDX) model of colon cancer with larger-sized POEG-co-PVD carrier as a control. PDX model has tumor heterogeneity and human stromal microenvironment, which is a good preclinical model to evaluate the biodistribution, penetration and therapeutic effect of drugs and formulations. **Figure 3C** shows the NIR images of PDX tumor-bearing NSG mice treated with DiR-loaded micelles. More DiR signals were observed in the tumors treated with DiR/PGEM compared to DiR/POEG-co-PVD-treated tumors at different time points (6 h, 12 h and 24 h). Tumors and major organs including heart (H), liver (Li), spleen (S), lung (Lu) and kidney (K) were harvested for *ex vivo* imaging at 24 h post-injection (**Figure 3D**). Larger amounts of DiR/PGEM signals were observed in the tumor tissues (T1 and T2) compared to other major organs. These results indicated that PGEM carrier was highly effective in mediating selective accumulation in the PDX model.

The PDX tumors treated with DiR-loaded PGEM and POEG-co-PVD nanoparticles were further frozen sectioned for penetration study. The slices in the tumor core were stained with DAPI for fluorescence imaging (**Figure 3E**). More DiR signals were observed in the tumor core treated with DiR-loaded PGEM nanoparticles than the one treated with DiR-loaded POEG-co-PVD nanoparticles, indicating better penetration capacity of PGEM carrier in the PDX tumor core.

Furthermore, we evaluated the penetration of PGEM carrier in PANC02 pancreatic cancer model. To minimize any individual differences in tumors, rhodamine and fluorescein were loaded into the PGEM and POEG-co-PVD carriers, respectively, as fluorescence probes. Then equal amounts of the two dye-loaded nanoparticles were mixed together and co-injected intravenously into the same mouse. As shown in **Figure 3F**, the green fluorescence signals from fluorescein/POEG-co-PVD nanoparticles were only weakly detected, whereas strong red fluorescence signals from rhodamine/PGEM nanoparticles were detected in the tumor's core. This result indicated a deeper penetration ability of the PGEM carrier. To rule out the possibility that different intensities of the two fluorophores were responsible for the differential intensities in the core, we switched the fluorescence probes, and used PGEM carrier to load fluorescein and POEG-co-PVD to load rhodamine. Similar to the conclusions derived from the experiments shown in **Figure 3F**, fluorescein-loaded PGEM showed higher tumor uptake and deeper tumoral diffusion than rhodamine-loaded POEG-co-PVD (**Figure 3G**).

### Efficacy against PANC02 tumor

**Figure 4:**
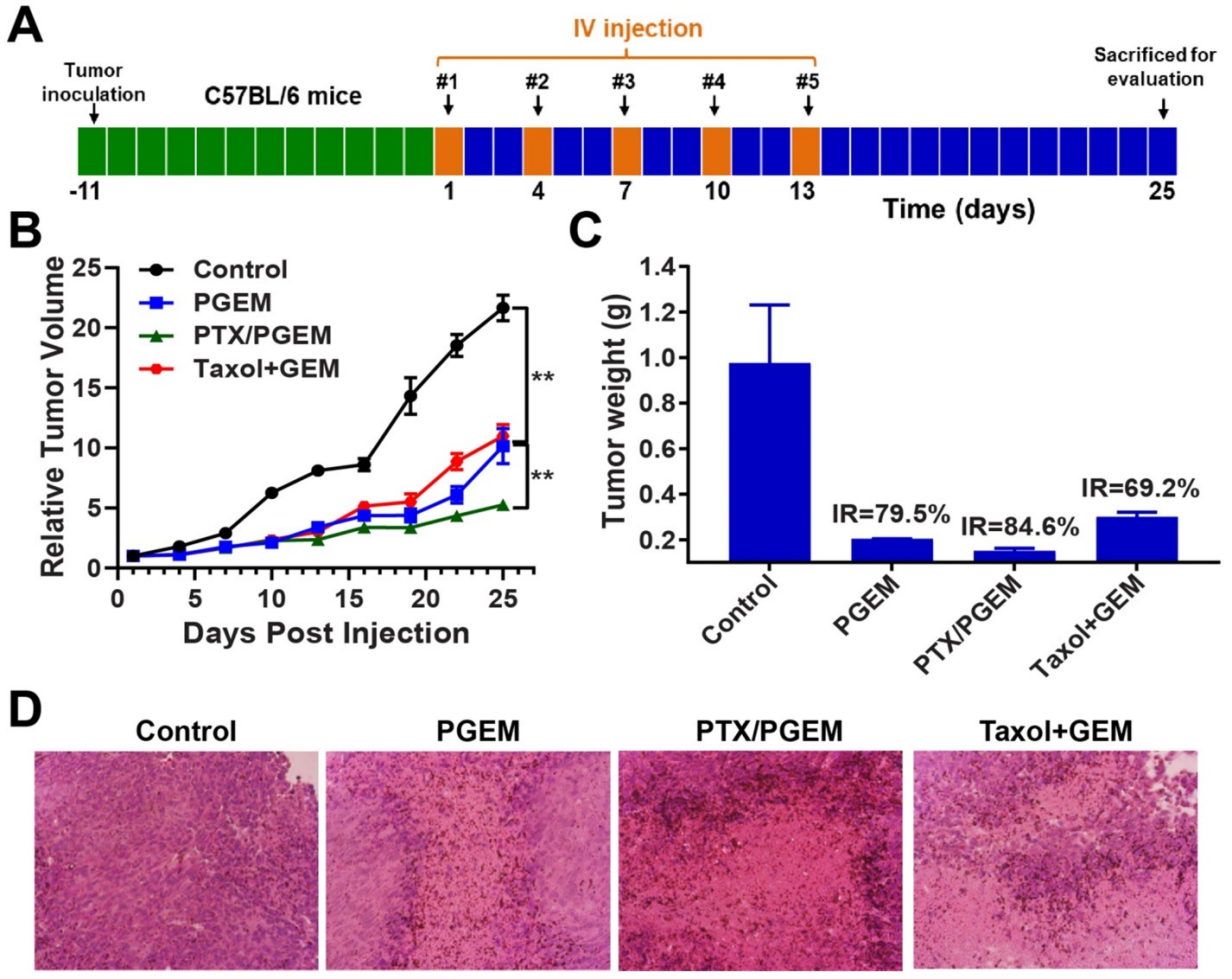
*In vivo* therapeutic effect in the PANC02 tumor model. **(A)** PANC02 cells were subcutaneously injected 12 days before treatment of various formulations, including saline, PGEM, PTX/PGEM and the mixture of Taxol and free GEM. Five intravenous injections were made every 3 days. **(B)** Relative tumor volume changes of the mice treated with various formulations. **(C)** Tumor weights of the mice receiving different treatments and tumor inhibition rate (IR) of various formulations. **(D)** Histological analyses of H&E stained tumor sections in each treatment group.

For the efficacy study, we initially examined the therapeutic efficacy of PTX/PGEM micelles in a murine pancreatic cancer model (PANC02). PANC02 is a unique tumor model with high resistance to virtually all clinically used chemotherapeutic agents, which could be used as a close mimic of human pancreatic cancer^32^, ^33^. PANC02 cells were subcutaneously injected into C57BL/6 mice. After 12 days, the tumor-bearing mice were intravenously (IV) injected with saline, PGEM carrier, PTX/PGEM, and Taxol combined with free GEM (**Figure 4A**).

PGEM prodrug micelles showed similar anti-tumor activity as the combination group of Taxol and free GEM (**Figure 4B**). PTX/PGEM micelles showed much higher anti-tumor activity than that of PGEM carrier. After sacrificing the mice, the tumor weights were measured (**Figure 4C**). The mice treated with PTX/PGEM micelles showed the lowest tumor weight and highest tumor inhibition rate (84.6%), which further confirmed its improved therapeutic efficacy over other formulations.

**Figure 4D** shows the hematoxylin and eosin (H&E)-stained images of tumor sections after various treatments. Large nuclei were observed in the tumor cells with saline treatment, while shrunk nuclei were observed in the tumor tissues with other treatments. Among them, the mice treated with PTX/PGEM showed the most significant necrotic damages of tumor tissues, further suggesting its best anti-tumor activity.

### The changes of PANC02 tumor immune microenvironment after various treatments

**Figure 5:**
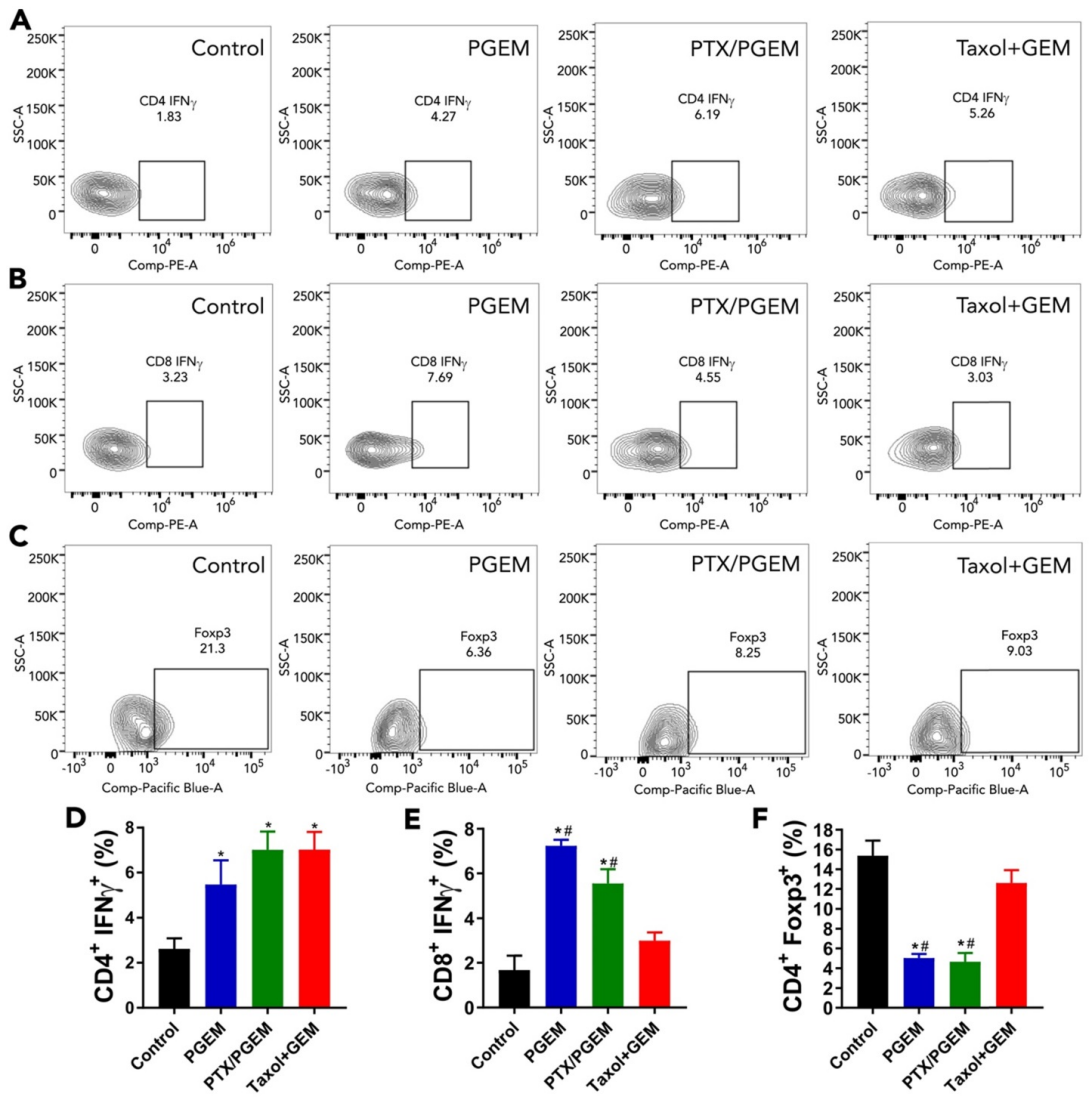
Flow cytometry analysis of immune cells in tumor tissues after treatment with various formulations. Representative flow cytometry gatings of tumor infiltrating immune cells, including CD4^+^IFNγ^+^ T cells **(A)**, CD8^+^IFNγ^+^ T cells **(B)** and CD4^+^ FoxP3^+^ Treg cells **(C)**. The percentage of tumor infiltrating immune cells was correspondingly quantified **(D-F)**. The results are reported as mean ± S.E.M. *p < 0.05, **p < 0.01 (vs control), ^#^*p* < 0.05 (vs Taxol+free GEM).

The changes in the PANC02 tumor immune microenvironment following various treatments were investigated by flow cytometric analysis of the immune cell populations in the tumors. As shown in **Figure S12**, there are no significant changes in the numbers of total CD4^+^ T cells in the tumors after various treatments compared with the control group. After treatment with PGEM, the relative numbers of CD8^+^ T cells in the tumors were significantly increased. IFN-γ is a powerful molecule produced by T cells, which plays an important role in eliminating solid tumors^34^. We found that the relative numbers of IFN-γ^+^ CD4^+^ T cells (**Figure 5A&D**) and IFN-γ^+^ CD8^+^ T cells (**Figure 5B&E**) in the tumors were significantly increased after treatment with PGEM and PTX/PGEM. Treg cells are a subtype of T cells that contribute to an immunosuppressive microenvironment. PGEM and PTX/PGEM treatments could significantly decrease the number of Treg cells in the tumor tissues (**Figure 5C&F**). It is noticed that PTX/PGEM group showed higher relative numbers of IFN-γ^+^ CD8^+^ T cells, and lower numbers of Treg cells compared with the combination group of Taxol and free GEM. Overall, our formulations could induce a more immunoactive tumor microenvironment, leading to an enhanced anti-tumor immune response.

We also compared the anti-tumor immunity mediated by POEG-*co*-PVD treatment with that mediated by PGEM treatment (**Figure S13**). PGEM treatment led to the higher relative numbers of CD8^+^ T cells (**Figure S13B**) and lower numbers of Treg cells (**Figure S13E**) in the tumors compared to POEG-*co*-PVD treatment, indicating an important role of GEM in increasing CD8^+^ T cells and depleting Treg cells in the tumors. It is also noted that compared to control group, both POEG-*co*-PVD and PGEM treatments led to increases in the numbers of IFN-γ^+^ CD4^+^ T (**Figure S13C**) and IFN-γ^+^ CD8^+^ T (**Figure S13D**) cells, and there is no significant difference between these two treatments. This suggested that the polymer backbone POEG-*co*-PVD contributed to part of anti-tumor immunity of the formulation. We also evaluated the toxicity of POEG-*co*-PVD backbone (**Figure S14**) at a dose that was 4 times higher than that used in therapy study. There was a slight increase in the body weights of the mice treated with POEG-*co*-PVD backbone, suggesting its excellent safety profile.

### Efficacy in other tumor models

**Figure 6:**
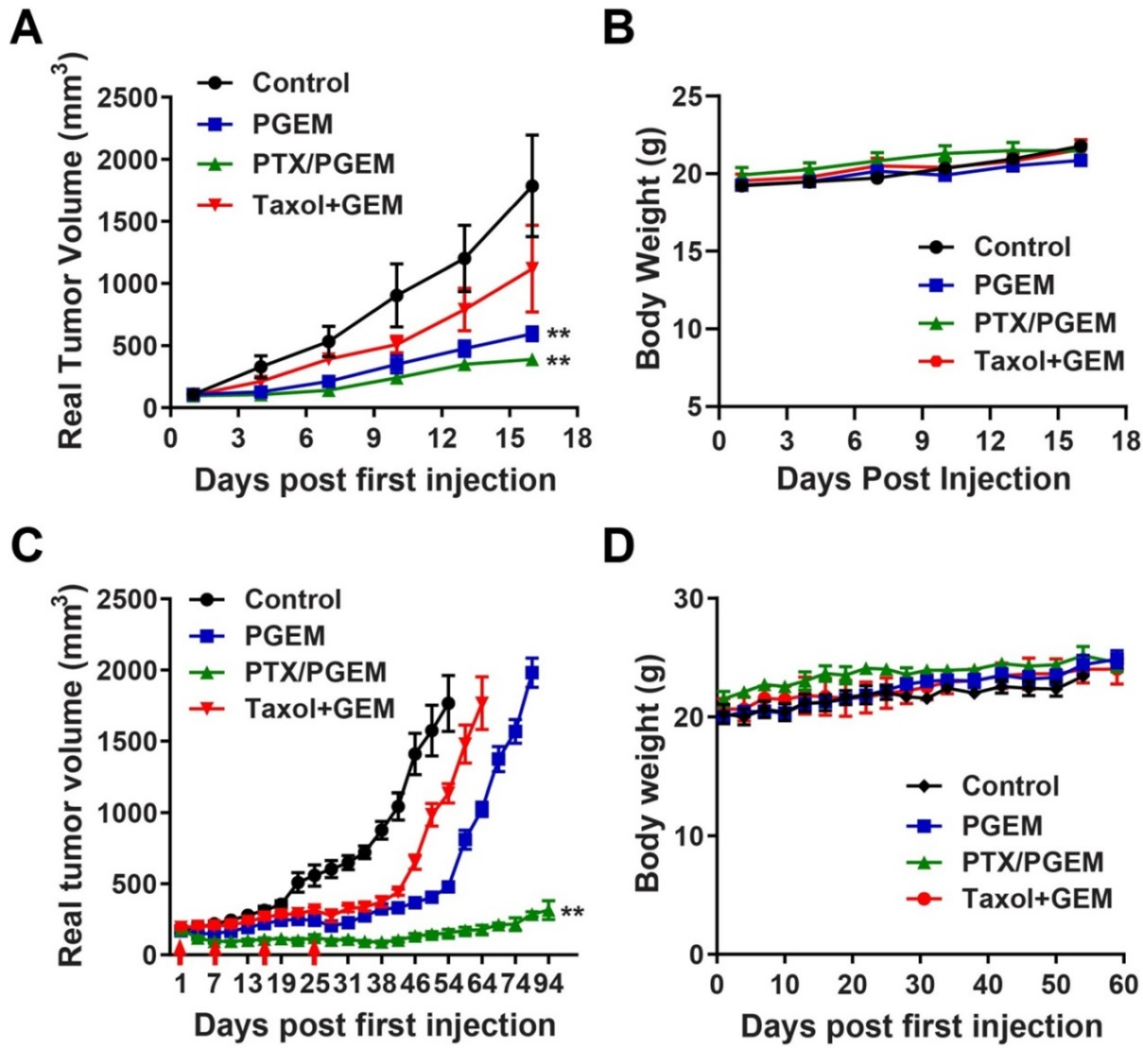
*In vivo* efficacy in other tumor models, including CT26 and PDX model. **(A)** Real tumor volume changes of the CT26 tumor-bearing mice treated with various formulations. **(B)** Body weights of the CT26 tumor-bearing mice after treatment. **(C)** Real tumor volume changes of the PDX tumor-bearing mice treated with various formulations. **(D)** Body weights of the PDX tumor-bearing mice after treatment. The results are reported as mean ± S.E.M. *p < 0.05, **p < 0.01 (vs Taxol+GEM)).

In addition to pancreatic cancer (PANC02) model, we also evaluated the efficacy of different formulations in other tumor models, including CT26 murine colon cancer model, and a PDX model of colon cancer. Compared to PANC02 model, CT26 tumor was more sensitive to the PGEM carrier: the antitumor activity of PGEM alone was significantly higher than that of combination of Taxol with free GEM (**Figure 6A**). Incorporation of PTX into PGEM further increased the antitumor activity in CT26 model. We further evaluated the therapeutic effects of various formulations in PDX model, which well recapitulates the histologic and molecular parameters of the human disease. **Figure 6C** and**Figure S8** show the real and relative tumor volume of mice treated with various formulations. Compared to Taxol/GEM combination, PGEM treatment could slow down the tumor growth. At day 64, the tumor treated with Taxol/GEM combination reached the volume limitation of ∼2000 mm^3^, while the mean tumor volume for PGEM-treated group is only 1024 mm^3^. At day 80, the tumors treated with PGEM reached the volume limitation of ∼2000 mm^3^** (Figure 6C)**. In comparison, PTX/PGEM micelles were more effective in inhibiting the tumor growth. The tumors shrank significantly after the first 2 injections and then became stabilized in sizes throughout the entire follow-up period. Even for 69 days after last treatment (day 25), the tumor volumes only increased by 0.7-fold compared to the initial tumors before first treatment **(Figure S8)**. In addition, there were no obvious changes in the body weights of the mice (**Figure 6B&D**), suggesting the low systemic toxicity of the formulations in these models.

## Discussion

Particle size is an important parameter of nanomedicines, which significantly affects their t1/2 in blood, tumor accumulation and the subsequent step of penetration 35-37. It has been reported that micelles of relatively large sizes (100~160 nm) demonstrated longer blood circulation time and higher tumor accumulation compared to the counterparts of smaller sizes, but less anti-tumor activity due to the poorer tumor penetration 9. Accumulating evidences have demonstrated that nanomedicines with small sizes (10 ~ 30 nm) exhibited superior tumor penetration and enhanced therapeutic efficacy, particularly for pancreatic cancer 7-9. To balance the tumor accumulation and penetration, tumor-triggered size transformable nanoparticles have been developed 38-40. However, the initial large size of these NPs prior to size transition may limit their extravasation into some tumors with small blood vessel pores. In this work, we report a novel tumor-permeable nanocarrier based on a GEM-conjugated polymer (PGEM). We discovered that conjugation of GEM to the POEG-co-PVD polymer backbone drastically reduced the nanoparticle size from 160 nm to 13 nm (**Figure 2B&C**). The small-sized PGEM carrier showed better tumor penetration than the larger POEG-co-PVD NPs, yet without compromises in the t1/2 in blood and tumor accumulation (**Figure 3**).

**Figure 7:**
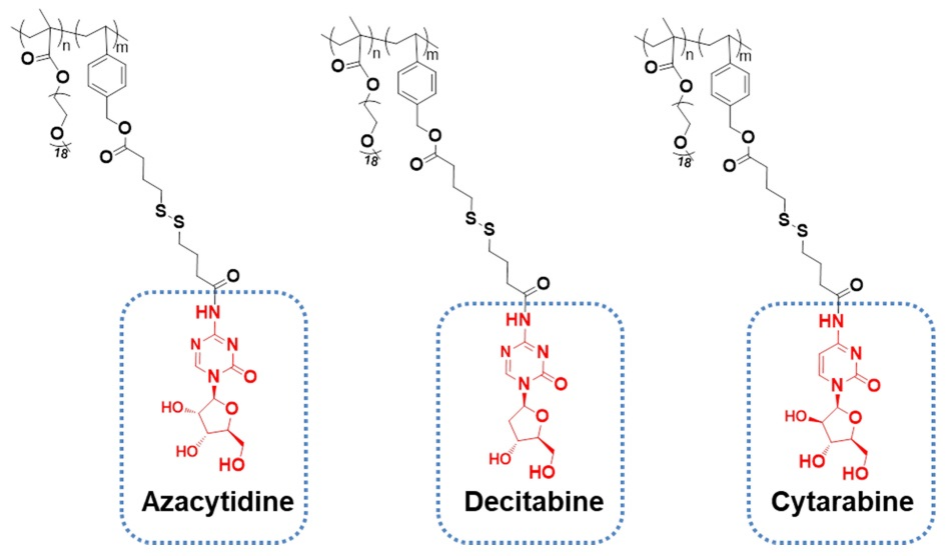
Structural analogues of PGEM polymers.

More importantly, PGEM carrier has overcome the limitations of small-sized polymeric carriers in drug loading. Generally, small polymeric micelles can be achieved by increasing the hydrophilic/hydrophobic block ratio 41, 42. However, at such a high ratio, a thick shell with a “tiny” core will be generated, leading to the low encapsulation capacity and poor stability of the drug-loaded micelles 43-46. Interestingly, the small PGEM carrier we developed was able to load/co-load a wide variety of hydrophobic agents such as curcumin, NLG919 and doxorubicin with excellent stability and high loading capacity, particularly for loading PTX, with a high capacity of 24 wt.%. Although many GEM-conjugated polymers have been developed, to our knowledge, the use of the GEM-conjugated polymer as a prodrug carrier to encapsulate other hydrophobic drugs has rarely been reported. This appears to be the first example of using a GEM-conjugated polymer as a small nanocarrier for co-delivery of hydrophilic drug GEM and hydrophobic agents. It should be noted that GEM can be readily replaced by many other nucleotides-based anticancer agents such as azacytidine, decitabine and cytarabine and the resulting new polymers showed similar properties of small sizes and high performance in loading hydrophobic drugs (**Figure 7**). Therefore, this carrier platform is not a GEM-specific system and can be extended to co-delivery of multiple distinct hydrophilic and hydrophobic agents for various combination therapies. Another advantage of our new platform lies in its simplicity with respect to the synthesis and formulation, which shall facilitate a rapid translation into clinic.

**Figure 8:**
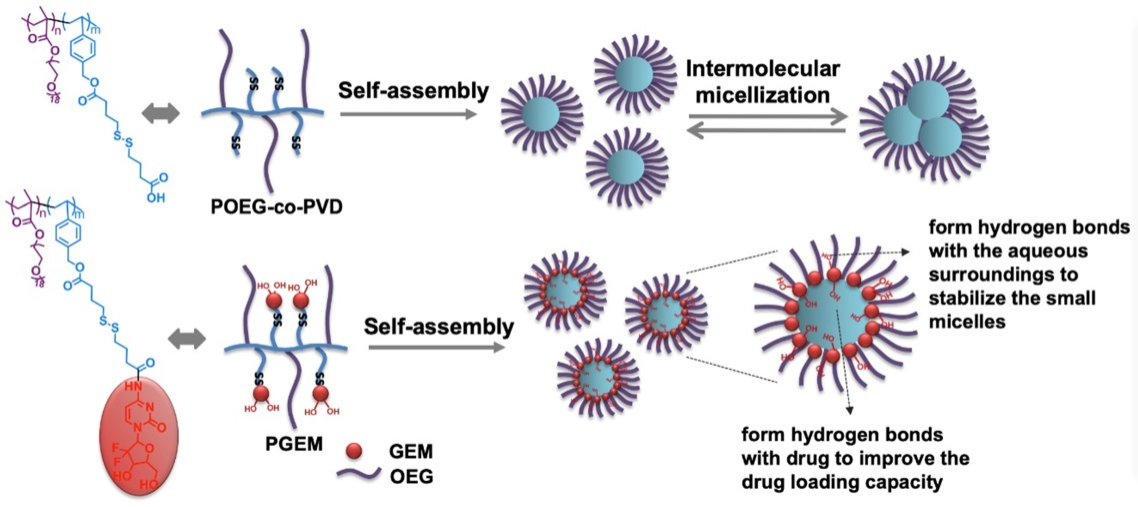
Schematic illustration of the self-assembly of small-sized nanoparticle by PGEM polymer and its improved drug loading capacity as a carrier due to hydrogen bonding.

Many amphiphilic polymers, such as POEG-*co*-PVD polymer without GEM motifs, tend to self-assemble into large micelles with a diameter of at least 100 nm. Paradoxically, after conjugation of GEM or other structural analogues, the particle size was significantly decreased, whereas the drug loading capacity and micelle stability improved. **Figure 8** shows our proposed model for the structural changes of POEG-*co*-PVD following conjugation with GEM. The formation of large micelles by POEG-co-PVD polymer might be explained by multimicelle aggregates (MMAs) mechanism, in which the small micelles formed at the initial stage are not stable enough and quickly flocculate and form larger multimicelle aggregates through intermicellar interactions 47. For the PGEM polymer, GEM molecules at the interface may form hydrogen bonds with the aqueous surroundings to stabilize the small micelles. This model could also explain why PGEM polymer has high drug loading capacity and excellent stability. Multiple benzyl ring and alkyl chain in the hydrophobic core could encapsulate hydrophobic agents through π-π stacking and hydrophobic interaction. Moreover, some hydroxyl groups from GEM that are oriented towards the micelle core may form hydrogen bonds with drugs to enhance the drug loading capacity and stability. More studies confirming this hypothesis are warranted in the future.

In addition to formulation advancement, this work has provided an effective regimen for improved anti-tumor immunity and enhanced cancer treatment. PTX/PGEM micelles exhibited excellent tumor inhibition effect in multiple tumor models, including PANC02 pancreatic cancer, CT26 colon cancer and clinically relevant PDX model (**Figure 6**), suggesting its great potential in clinical translation. The high therapeutic efficacy of PTX/PGEM micelles is likely attributed to both excellent tumor accumulation and effective penetration of the nanocarrier and the synergistic chemotherapeutic effect of the co-delivered PTX and GEM. In addition, the immunostimulatory effect of PGEM carrier shall also play a role. We found the small PGEM carrier could boost anti-tumor immune response with fewer immunosuppressive Treg cells and more production of IFN-γ (**Figure 5**). It is also noted that PTX/PGEM shows better effect in activating the immune system compared with Taxol+GEM combination, which is probably due to a more effective delivery of PTX and GEM into the tumors via the small-sized carrier.

## Conclusions

We developed a small-sized carrier platform based on a new GEM-conjugated polymer that was able to load a wide variety of hydrophobic drugs with high loading capacity and excellent stability. PGEM nanocarrier could efficiently accumulate and penetrate into the tumor core and inhibit tumor growth. In addition, it improved the antitumor immunity by increasing IFN-γ^+^ CD4^+^ and IFN-γ^+^ CD8^+^ T cells and decreasing Treg cells. Incorporation of PTX into the PGEM carrier further improved the anti-tumor effect in multiple tumor models through synergistic action with the co-delivered GEM, which demonstrated the wide application of the new regimen in cancer treatment.

## Figures and Tables

**Table 1:** Characterization of PGEM micelles loaded with PTX at different carrier/drug ratios.

Micelles	Mass ratio (mg: mg)	Size (nm)*^a^*	PDI*^b^*	DLC(%)*^c^*	DLE(%)*^d^*
**PGEM**	--	13.14	0.169		
**PGEM: PTX**	20: 1	13.95	0.136	4.4	93.8
**PGEM: PTX**	10: 1	14.90	0.151	8.3	91.3
**PGEM: PTX**	5: 1	17.77	0.227	14.8	88.5
**PGEM: PTX**	2.5: 1	23.07	0.265	24.2	84.6

Notes: ^a^Measured by dynamic light scattering particle sizer. ^b^PDI = polydispersity index. ^c^DLC = drug loading capacity. ^d^DLE = drug loading efficiency.

## Abbreviations

GEM: gemcitabine
PTX: paclitaxel
OEG: oligo(ethylene glycol) methacrylate
PGEM: POEG-co-PVDGEM
PDX: patient derived xenograft model
NPs: nanoparticles
CDA: cytidine deaminase
DLC: drug loading capacity
DLE: drug loading efficiency
PDI: polydispersity index
AIBN: 2, 2-Azobis(isobutyronitrile)
MTT: 3-(4,5-dimethylthiazol-2-yl)-2,5-diphenyl tetrazolium bromide
DMEM: Dulbecco's Modified Eagle's Medium
HOBT: 1-hydroxybenzotriazole
EDC: 1-(3-dimethylaminopropyl)-3-ethylcarbodiimide
DIPEA: Diisopropylethylamine
DOX·HCl: Doxorubicin hydrochloride salt
FBS: Fetal bovine serum
NMR: Nuclear magnetic resonance
FTIR spectroscopy: Fourier-transform infrared spectroscopy
GPC: gel permeation chromatography
DLS: dynamic light scattering
TEM: transmission electron microscopy
HPLC: high performance liquid chromatography
PBS: phosphate-buffered saline
FBS: fetal bovine serum
CI: combination index
DiR: 1,1'-Dioctadecyl-3,3,3',3'-Tetramethylindotricarbocyanine Iodide
GSH: reduced glutathione
CMC: critical micelle concentration
NIR: near-infrared fluorescent
IV: intravenously
