# Supplementary material for: High Loading of Hydrophobic and Hydrophilic Agents via Small Immunostimulatory Carrier for Enhanced Tumor Penetration and Combinational Therapy
Source: Theranostics. 2020 Jan 1;10(3):1136–50. doi: 10.7150/thno.38287 (PMC6956803; doi:10.7150/thno.38287)
Supplement: Supplementary file 1 — Supplementary figures and tables. [file thnov10p1136s1.pdf]

Supplementary Information

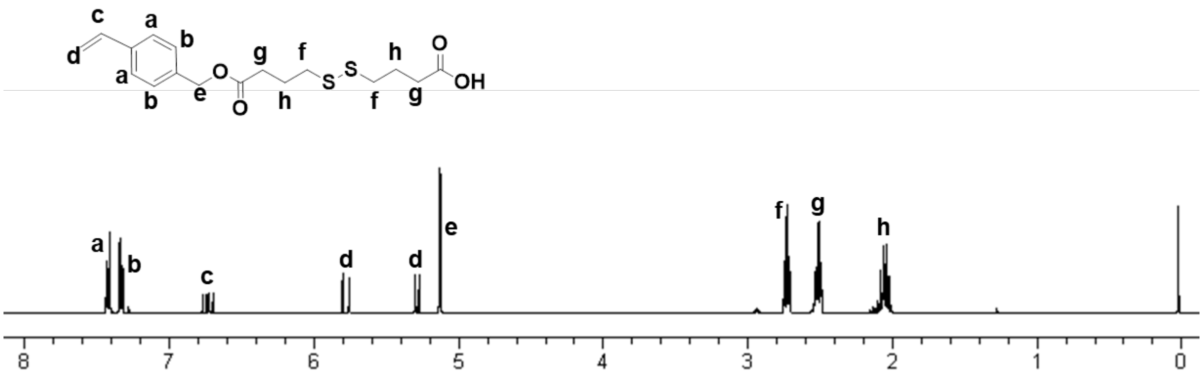

Figure S1 ^{1}H NMR spectrum of VD monomer in $CDCl_3$.

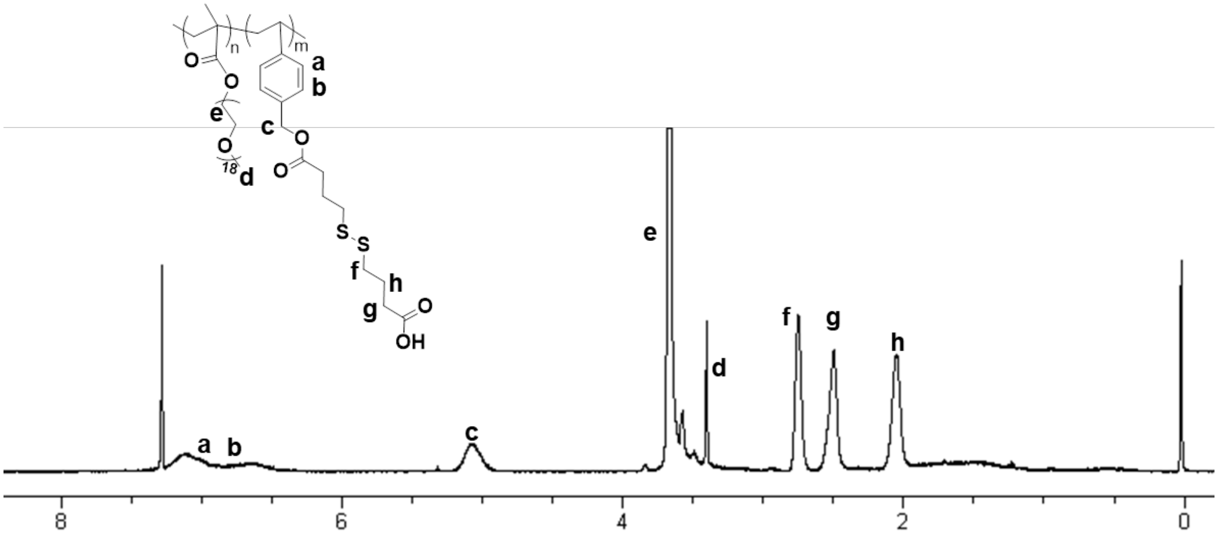

Figure S2 ^{1}H NMR spectrum of POEG-*co*-PVD polymer in $CDCl_3$.

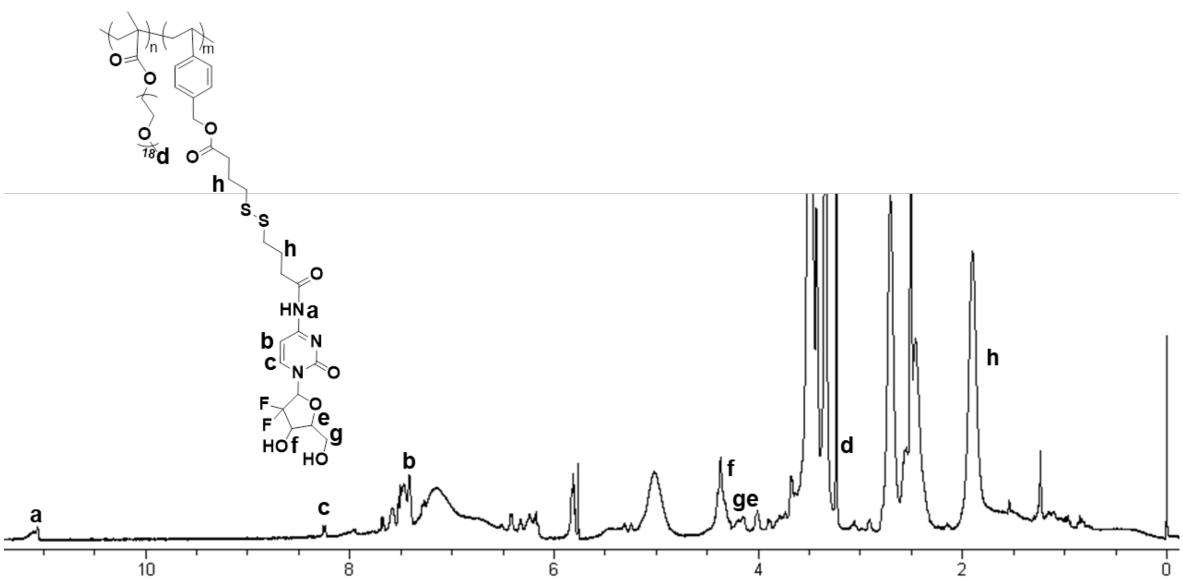

Figure S3 ^{1}H NMR spectrum of POEG-*co*-PVDGEM (PGEM) polymer in DMSO-d_6.

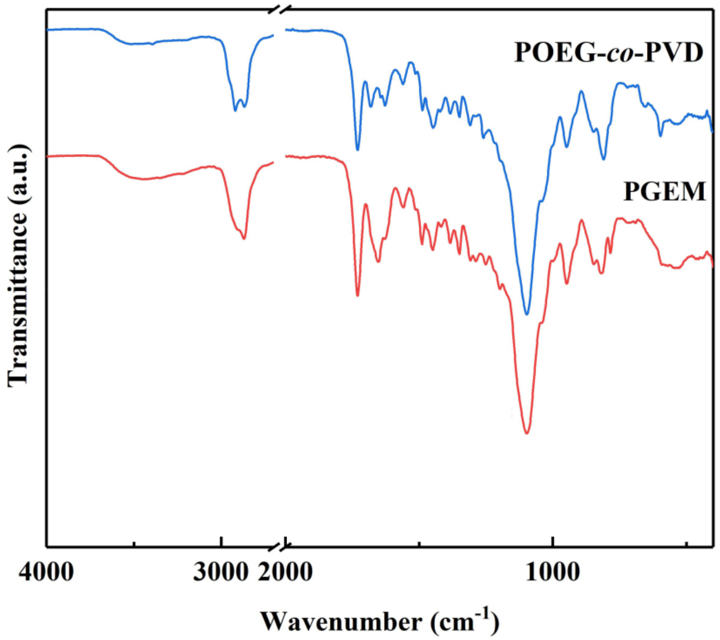

Figure S4 FTIR spectra of POEG-*co*-PVD and PGEM polymers in DMSO-d_6.

Table S1. Characterizations of the POEG-*co*-PVD and PGEM copolymers.

Polymers	OEG units	VD units	GEM units	M_n[a] (NMR)	M_n[b] (GPC)	M_w/M_n[b] (GPC)
POEG-*co*-PVD	9	23	0	15810	11600	1.13
PGEM	9	23	8	17910	9200	1.15

Notes: [a] Measured and calculated by NMR. [b]Measured by GPC with THF as the eluent, and the molecular weights and their distribution were calculated with polystyrene standards.

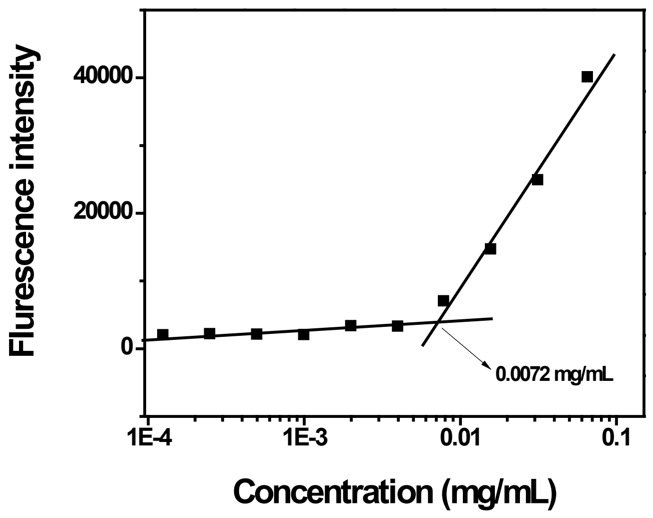

Figure S5 CMC value of PGEM micelle measured by fluorescence spectrometry using nile red as a fluorescence probe.

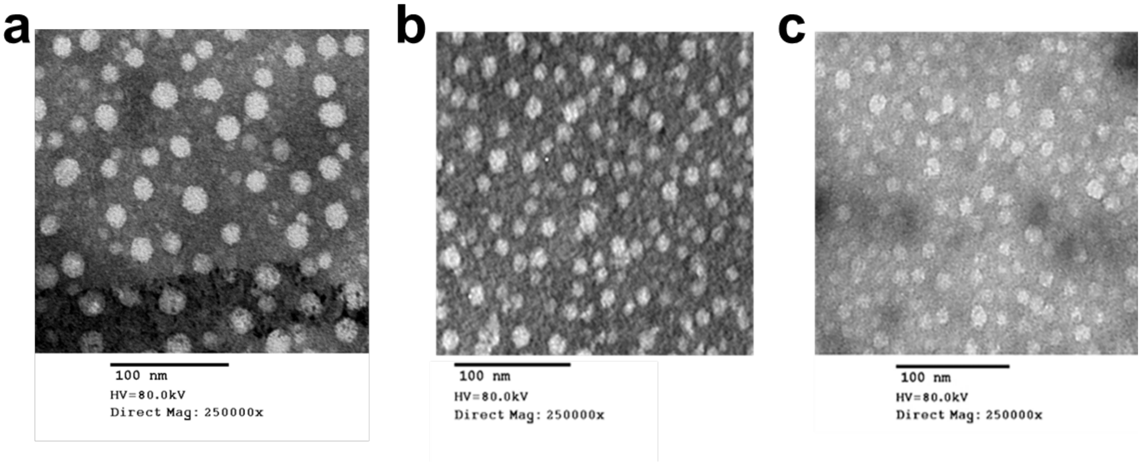

Figure S6 TEM images of POEG-co-PVD (a), PGEM (b) and PTX-loaded PGEM micelles (c).

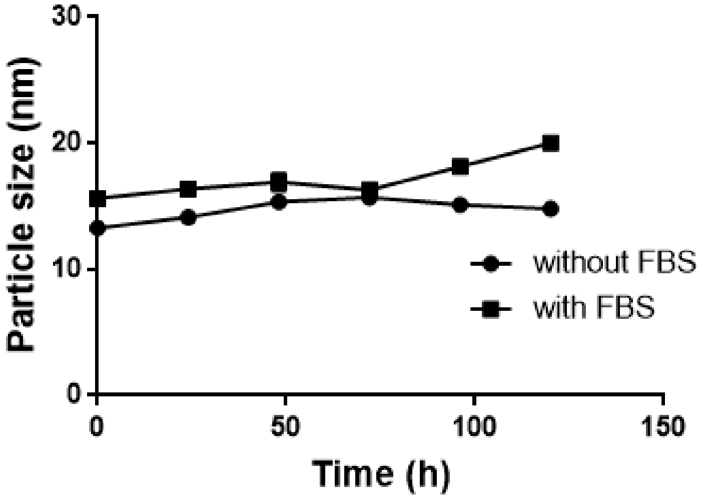

Figure S7. Stability of PTX-loaded PGEM NPs in PBS/FBS (1:1) stored at 4 °C. Values reported are the means ± SD for triplicate samples.

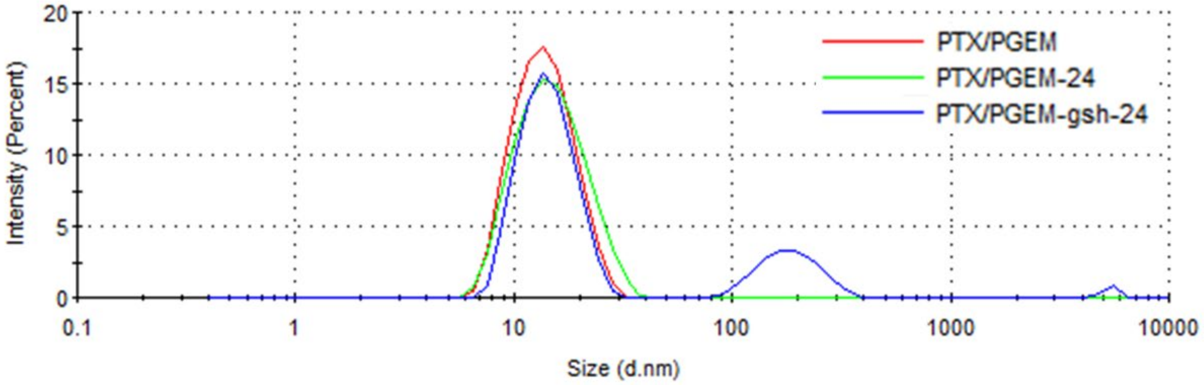

Figure S8. Size profiles of PTX/PGEM micelles in the presence/absence of GSH for 24 h.

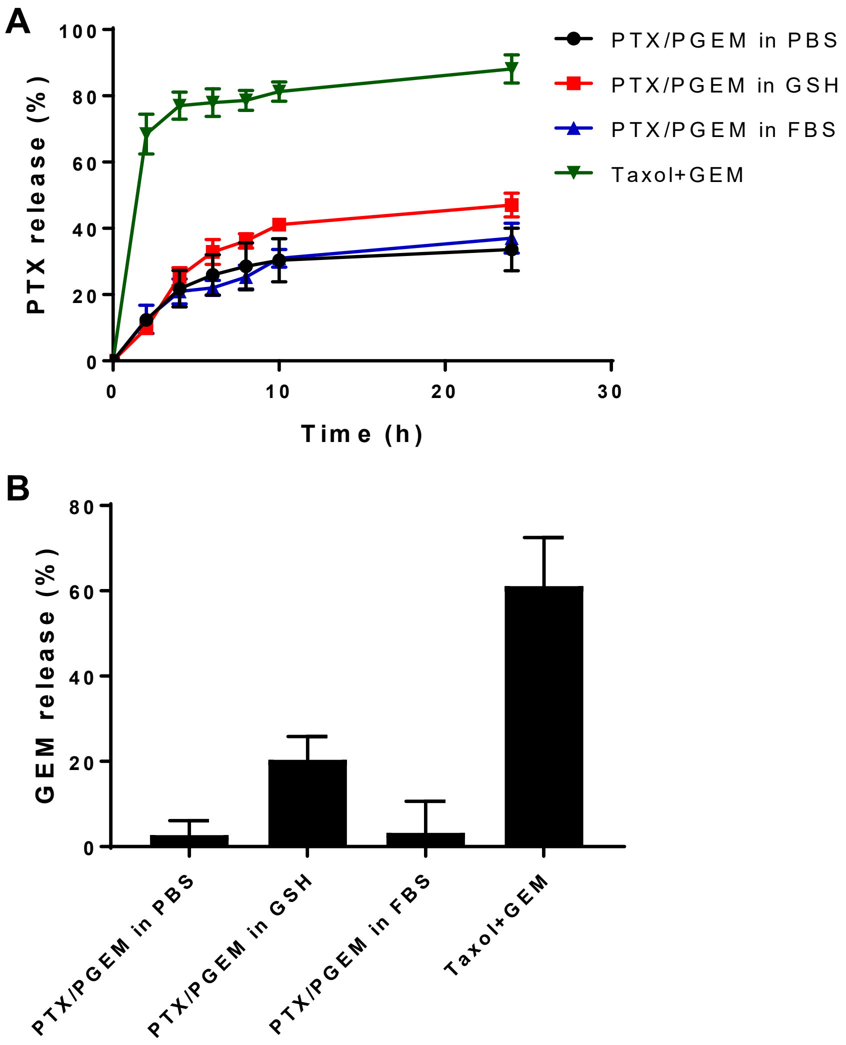

Figure S9. Release of PTX (A) and GEM (B) from PTX/PGEM micelles and Taxol plus GEM group in pH 7.4, pH 7.4+FBS and pH 7.4+ GSH environment.

Table S2. IC50 of GEM and PGEM in PANC02 and H7 cells.

Group	IC50 in PANC02	IC50 in H7
GEM	89.56 ng/mL	13.7 ng/mL
PGEM	265.1 ng/mL	88.66 ng/mL

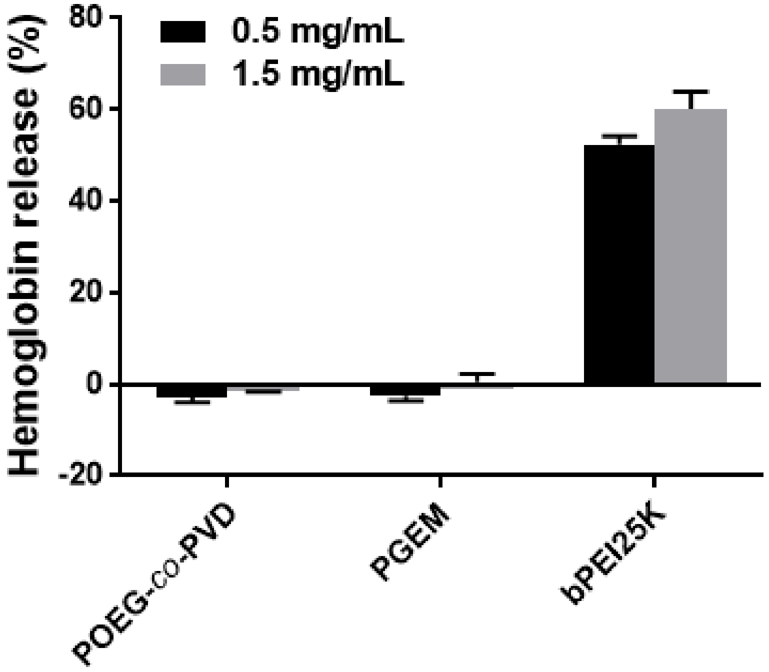

Figure S10. In vitro hemolysis assay of POEG-*co*-PVD and PGEM with branched PEI25K as a control. Values reported are the means ± SD for triplicate samples.

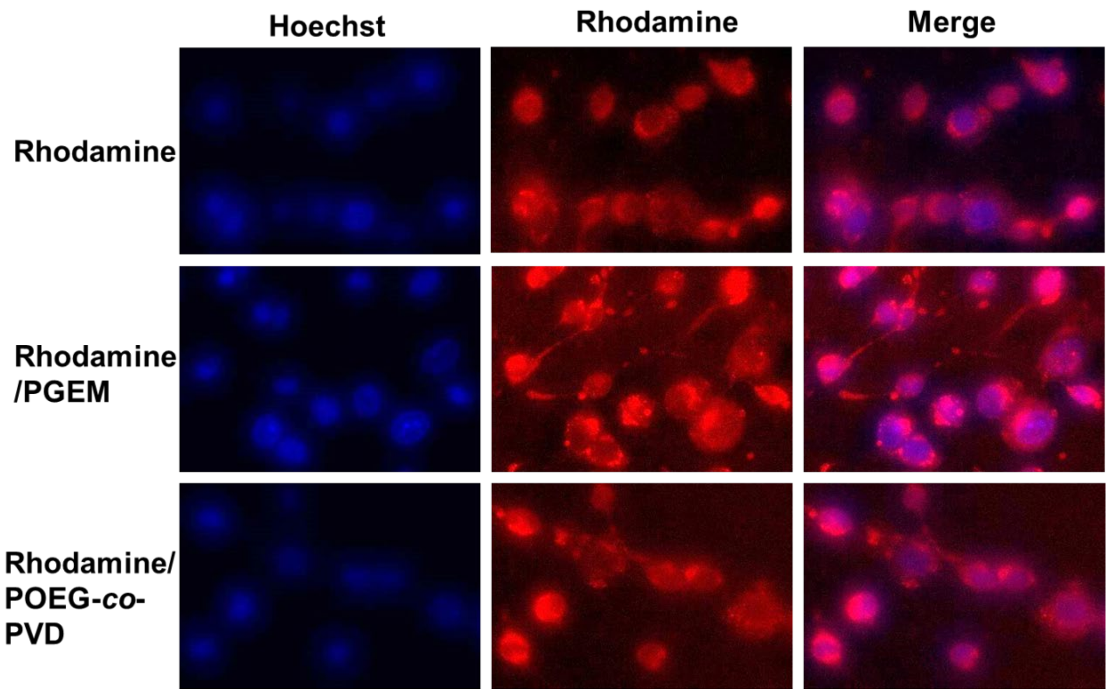

Figure S11. In vitro cellular uptake of rhodamine-loaded POEG-*co*-PVD and PGEM NPs with free rhodamine as a control. The PANC02 cells were incubated with various NPs for 4 h before fluorescence imaging.

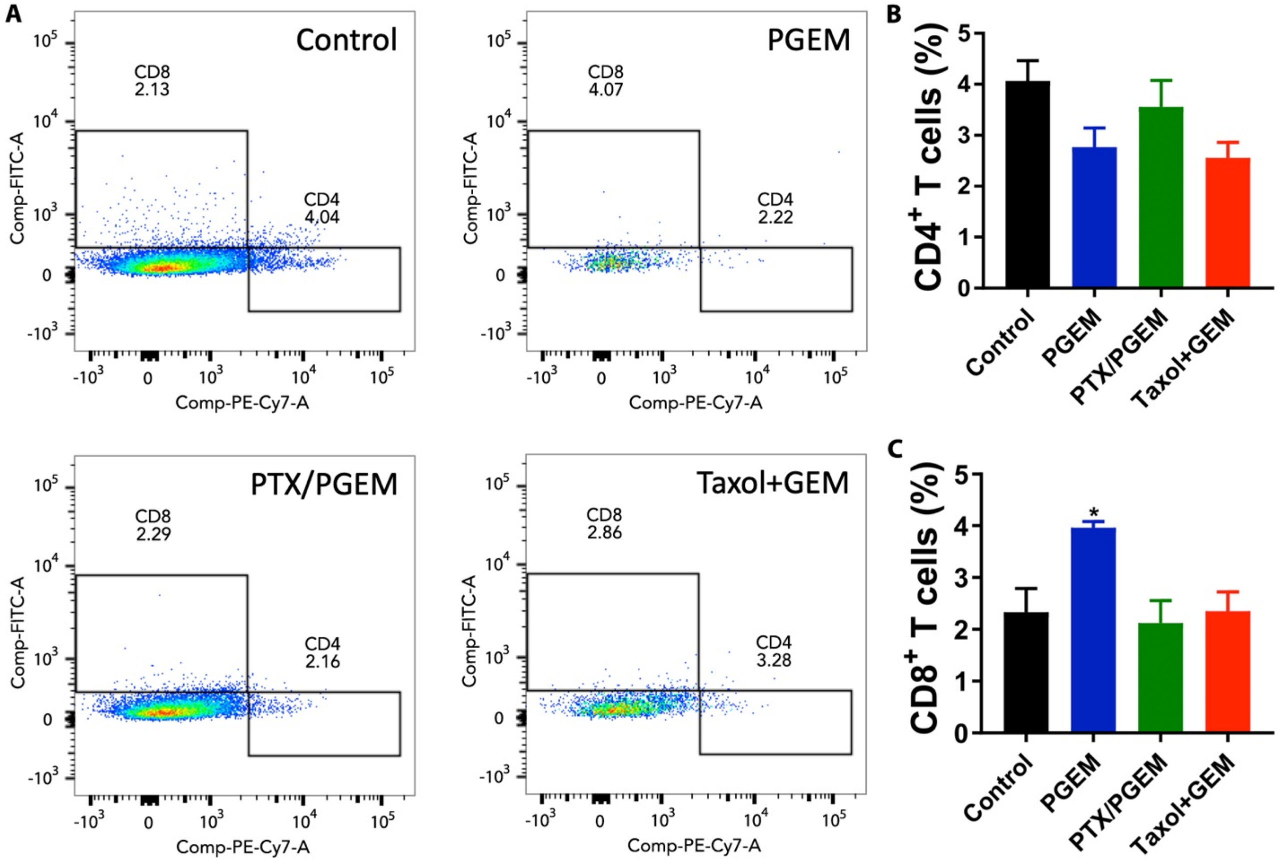

Figure S12. Representative flow cytometry gatings of tumor infiltrating T cells after treatment with various formulations (A). The percentage of $CD4^{+}$ T cells (B) and $CD8^{+}$ T cells (C) was quantified, and the results are reported as mean ± S.E.M. $^{*}p < 0.05$ (vs control), $^{\#}p < 0.05$ (vs Taxol+free GEM), $^{\&}p < 0.05$ (vs PTX/PGEM).

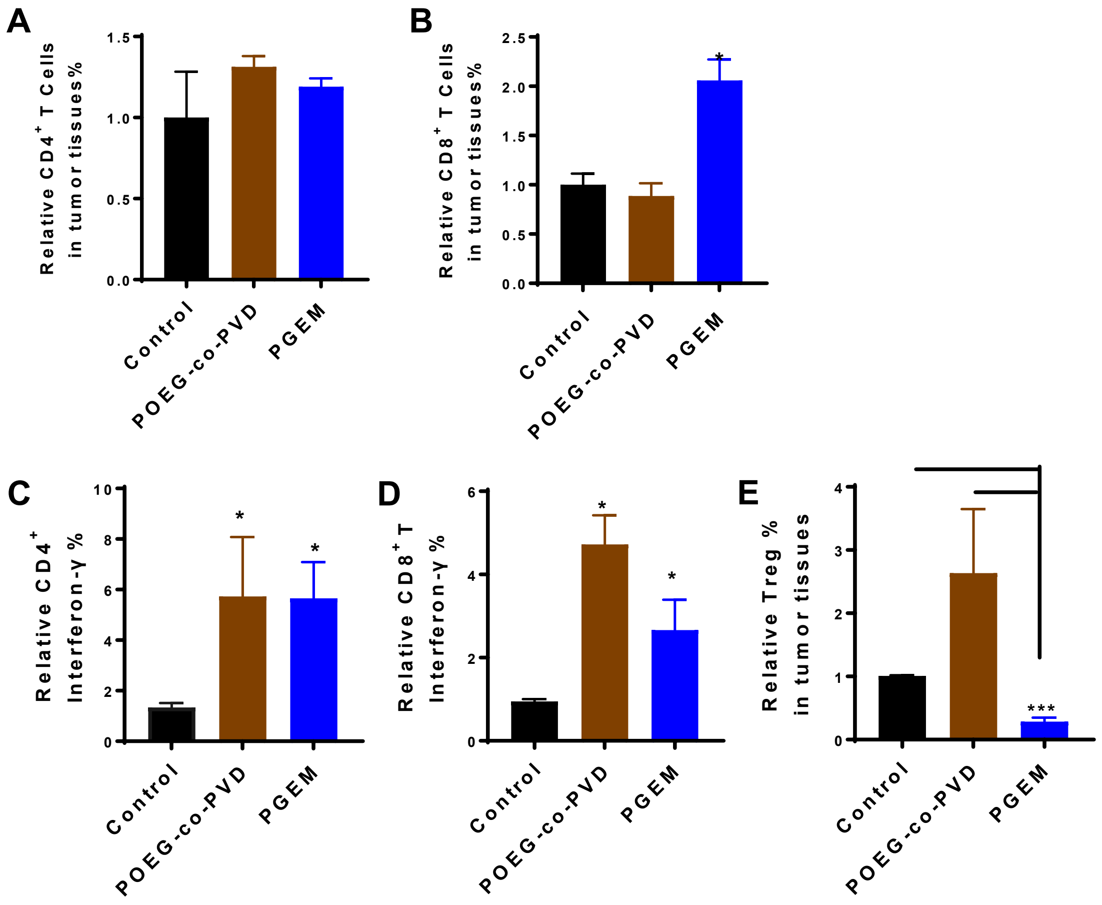

Figure S13. The percentage of tumor infiltrating immune cells, including CD4$^+$ T cells (A), CD8$^+$ T cells (B), CD4$^+$IFNγ$^+$ T cells (C), CD8$^+$IFNγ$^+$ T cells (D) and CD4$^+$ FoxP3$^+$ Treg cells (E), was quantified.

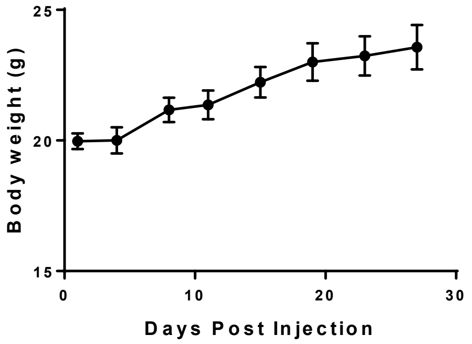

Figure S14. Body weight of POEG-*co*-PVD-treated mice.

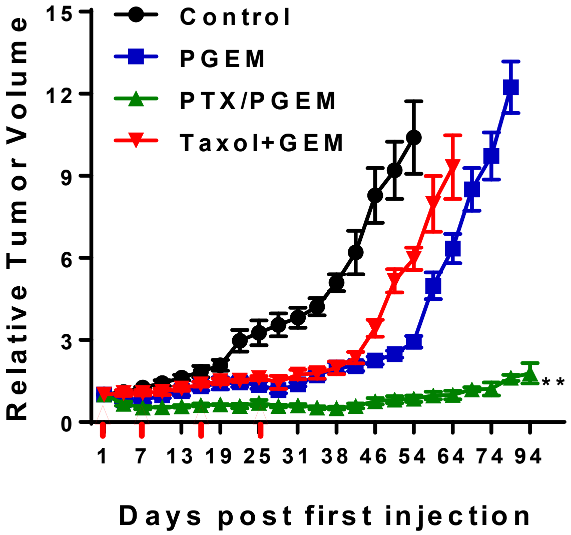

Figure S15. Relative tumor volume changes of the PDX tumor-bearing mice treated with various formulations. The results are reported as mean ± S.E.M. $^{**}p < 0.01$ (vs Taxol+GEM).
